# I’LL TELL YOU IF YOU ASK: MESSAGES ABOUT SUPPORT FROM AFRICAN AMERICAN WOMEN CAREGIVERS

**DOI:** 10.1093/geroni/igae098.1104

**Published:** 2024-12-31

**Authors:** Colleen Galambos, Abiola Keller, Katherine Platt, Jennifer Vidal

**Affiliations:** University of Wisconsin Milwaukee, Milwaukee, Wisconsin, United States; Marquette University, Milwaukee, Wisconsin, United States; University of Wisconsin Milwaukee, Milwaukee, Wisconsin, United States; University of Wisconsin Milwaukee, Milwaukee, Wisconsin, United States

## Abstract

Family caregivers are vital for ensuring the health of a growing older adult population. African American women represent a critical population of caregivers, since 60 percent of caregiving among older adults within the African American community is performed by women (Centers for Disease Control, 2024). Additionally, there is added cultural pressure to provide this care to family members and African American women caregivers (AAWC) face multiple competing stressors and demands (Imoh & Charity, 2023). Given these cultural dynamics, it is important to understand what type of assistance AAWC need to enable them to engage in self-care and support themselves in their caregiving role. Furthermore, there may be barriers faced by AAWC in accessing supportive interventions and resources. To increase knowledge about barriers and preferences for interventions and services, qualitative interviews were conducted with 12 AAWC of older adults. Interviews were audio recorded and transcribed using Microsoft TEAMS. To analyze the data, inductive content analysis was employed, responses were coded and emerging patterns determined. This presentation reports on the most frequently mentioned barriers and enablers to receiving support that emerged from the interviews as well as cultural considerations. Some barriers that surfaced from the data include supporting others over self, time constraints and logistical barriers. Some enablers or facilitators include sharing care responsibilities, support of family and friends, and obtaining information/receiving resources. Additional barriers and enablers and other experiences identified by the participants will be discussed. Community stakeholder’s response to the study will be highlighted.

